# A Rare Case of Traumatic Orbital Pseudomeningocele

**DOI:** 10.7759/cureus.77881

**Published:** 2025-01-23

**Authors:** Sophia Mirkin, Jeet Patel, Wen Wang, Corey Engel

**Affiliations:** 1 Dr. Kiran C. Patel College of Osteopathic Medicine, Nova Southeastern University, Fort Lauderdale, USA; 2 Neuroradiology, Mayo Clinic, Jacksonville, USA; 3 Radiology, University of Florida Health, Jacksonville, USA

**Keywords:** cerebrospinal fluid leak, imaging, orbital pseudomeningocele, orbital roof fracture, traumatic injury

## Abstract

A traumatic orbital pseudomeningocele is a rare complication of orbital trauma associated with orbital roof fractures and is underreported in the literature. Clinical signs may include proptosis, ophthalmoplegia, and diplopia, and when severe, there can be additional periorbital swelling from the anterior subcutaneous extension of the pseudomeningocele. We present a rare case of an orbital roof fracture resulting in an orbital pseudomeningocele with periorbital extension. CT is the preferred imaging modality for detecting orbital and other maxillofacial fractures, and it is sensitive to structural complications of this nature. In most cases, surgical repair is necessary. Our case emphasizes the significance of diagnostic imaging and collaborative care for optimal patient outcomes.

## Introduction

Orbital roof fractures are commonly the result of high-impact trauma, such as physical assaults, falls, and motor vehicle accidents [1,2]. There are several types of orbital roof fractures, including blow-in and blow-out fractures, both of which involve different mechanisms of injury [3,4]. The orbital roof is part of the anterior skull base, and fractures of the orbital roof are, therefore, often associated with other craniofacial injuries. These injuries include fractures of the frontal sinuses, orbital rims and orbital walls, naso-orbital-ethmoid (NOE) complex fractures, and LeFort fractures [2]. Additionally, there may be intracranial complications, including the presence of a cerebrospinal fluid (CSF) leak.

Skull base fractures may lacerate the dura and create the potential for a CSF leak [5]. This leak may result in the formation of a pseudomeningocele, an abnormal extradural collection of CSF secondary to a defect in the dura, and these can be associated with the formation of a fibrous capsule [6]. When CSF leaks into and forms a collection in the orbit, it is referred to as a traumatic orbital pseudomeningocele, which should be assessed for on CT whenever there is a displaced orbital roof fracture defect present [7]. Traumatic orbital pseudomeningoceles are rare and scarcely reported in the literature, primarily documented in isolated case reports [8-11]. We present a rare case of an orbital roof fracture resulting in an orbital pseudomeningocele with periorbital extension.

## Case presentation

**Figure 1:**
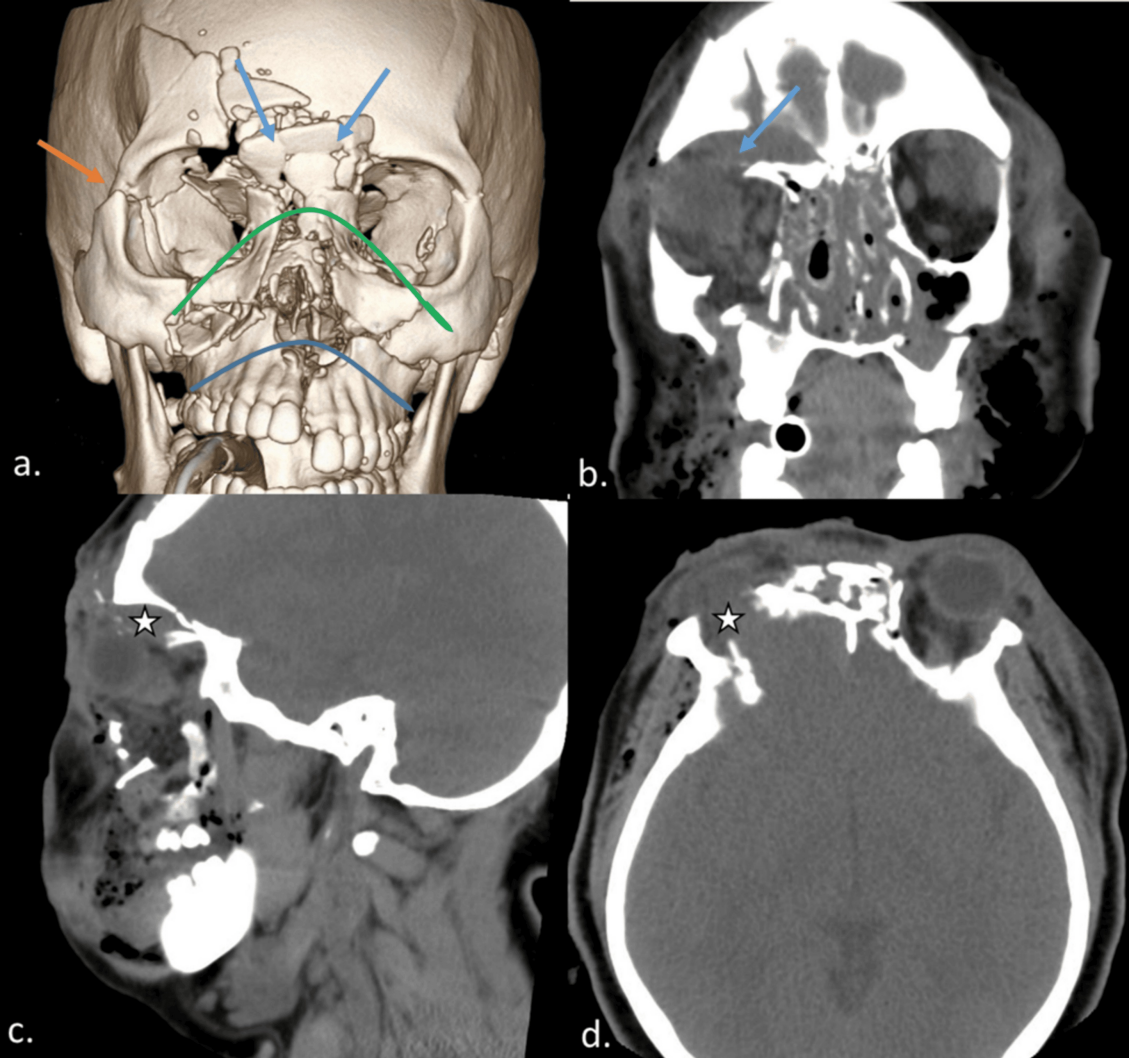
Initial CT at presentation The 3D volume-rendered image (a) demonstrates displaced fractures of the inferior maxillary sinus walls and nasal cavity walls following a LeFort I pattern (curved blue line), as well as displaced fractures of the bilateral medial orbital walls and orbital floors following a LeFort II pattern (curved green line). The right lateral orbital wall is also fractured (orange arrow). Along the central aspect, there are NOE fractures (blue arrows). On coronal reformatted CT soft tissue window images (b), a blow-in fracture of the right orbital roof with a wide fracture defect is apparent (blue arrow). Sagittal plane (c) and axial plane (d) images demonstrate the bulging of the dura (stars) into the superior right orbit through the fracture defect. CT: computed tomography, NOE: naso-orbital-ethmoid

A 32-year-old female was admitted after a severe motor vehicle accident in which she was unrestrained. The clinical examination revealed marked craniofacial deformities. CT of the head and maxillofacial region demonstrated extensive right orbital fractures with blow-in fracture of the orbital roof, causing downward bulging of the dura and blow-out fractures of the medial orbital wall and orbital floor. Additionally, there were comminuted bifrontal sinus wall fractures, bilateral LeFort I (horizontal maxillary) and II (pyramidal) fractures, NOE complex fractures, and right zygomaticomaxillary complex (ZMC) fractures (Figure 1).

**Figure 2:**
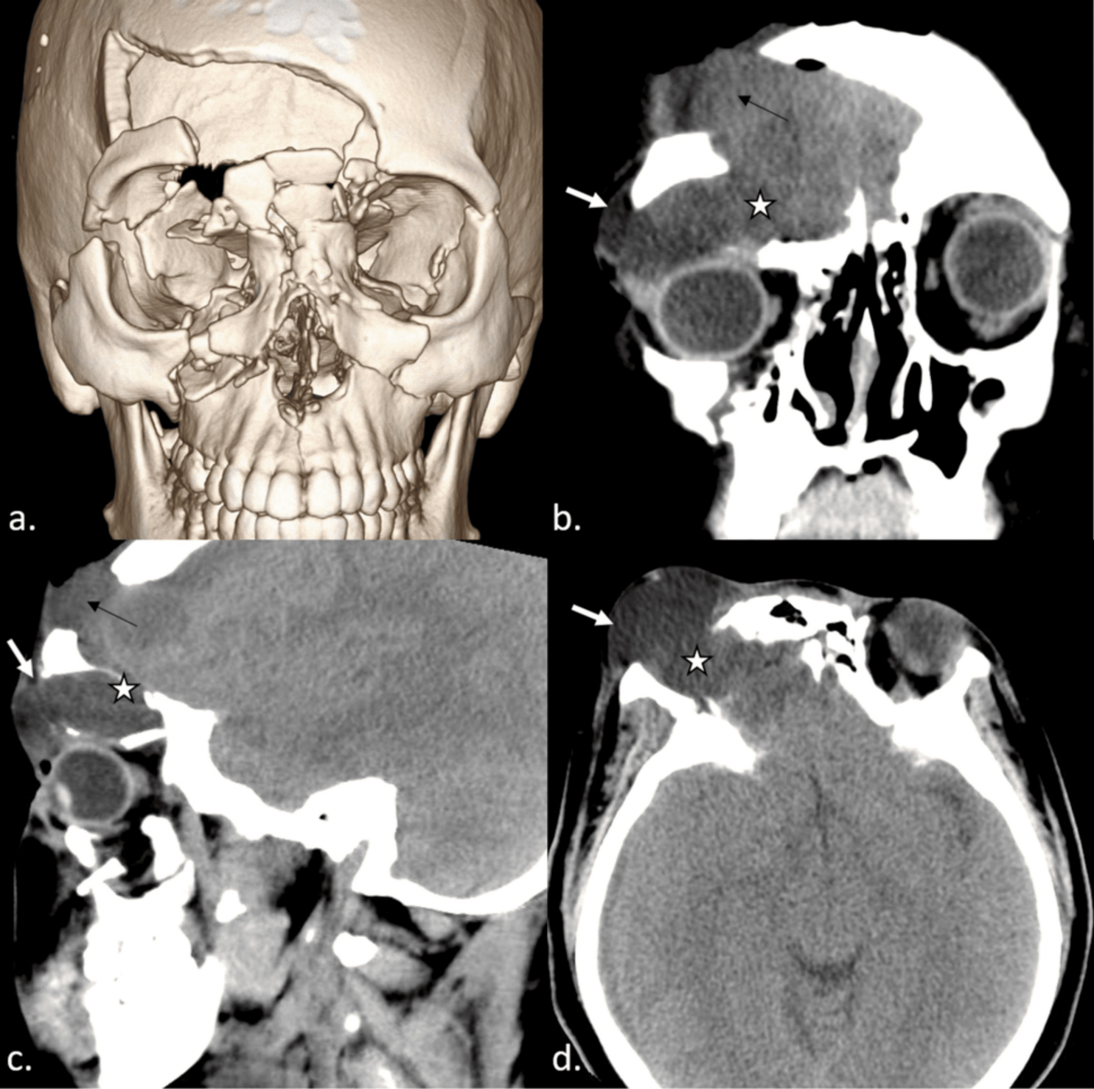
Follow-up CT images during hospital admission The 3D volume-rendered image (a) demonstrates changes from interval frontal cranioplasty. Coronal, sagittal, and axial plane images (b, c, d) demonstrate the development of a large extra-axial fluid collection occupying the right superior orbital space and extending into the right superior periorbital region (white arrows in b, c, and d) where it causes conspicuous bulging of the skin surface; this fluid collection also displaces the globe inferiorly. This collection originates from the intracranial compartment through the right orbital roof fracture defect (stars in b, c, and d) consistent with a pseudomeningocele. Coronal and sagittal images (b and c) also demonstrate an additional area of a pseudomeningocele (black arrows) extending through the right frontal calvarial defect into the forehead scalp. CT: computed tomography, 3D: three dimensional

A lumbar drain was placed by the interventional radiology (IR) service soon after admission to address a CSF leak from the extensive anterior skull base fractures. A flexible catheter was inserted into the lower back to remove CSF from the spinal canal, thereby regulating its pressure and preventing excess fluid buildup that can cause complications like increased pressure on the brain. The lumbar drain became accidentally dislodged approximately two weeks later. The patient then developed a noticeable bulge along the right periorbital region. CT maxillofacial examination was repeated, revealing a large pseudomeningocele extending from the anterior fossa into the right orbit and extending further into the right superior periorbital tissues, causing a mass effect on the right ocular globe with inferior displacement (Figure 2). IR then performed a prompt lumbar drain replacement.

**Figure 3:**
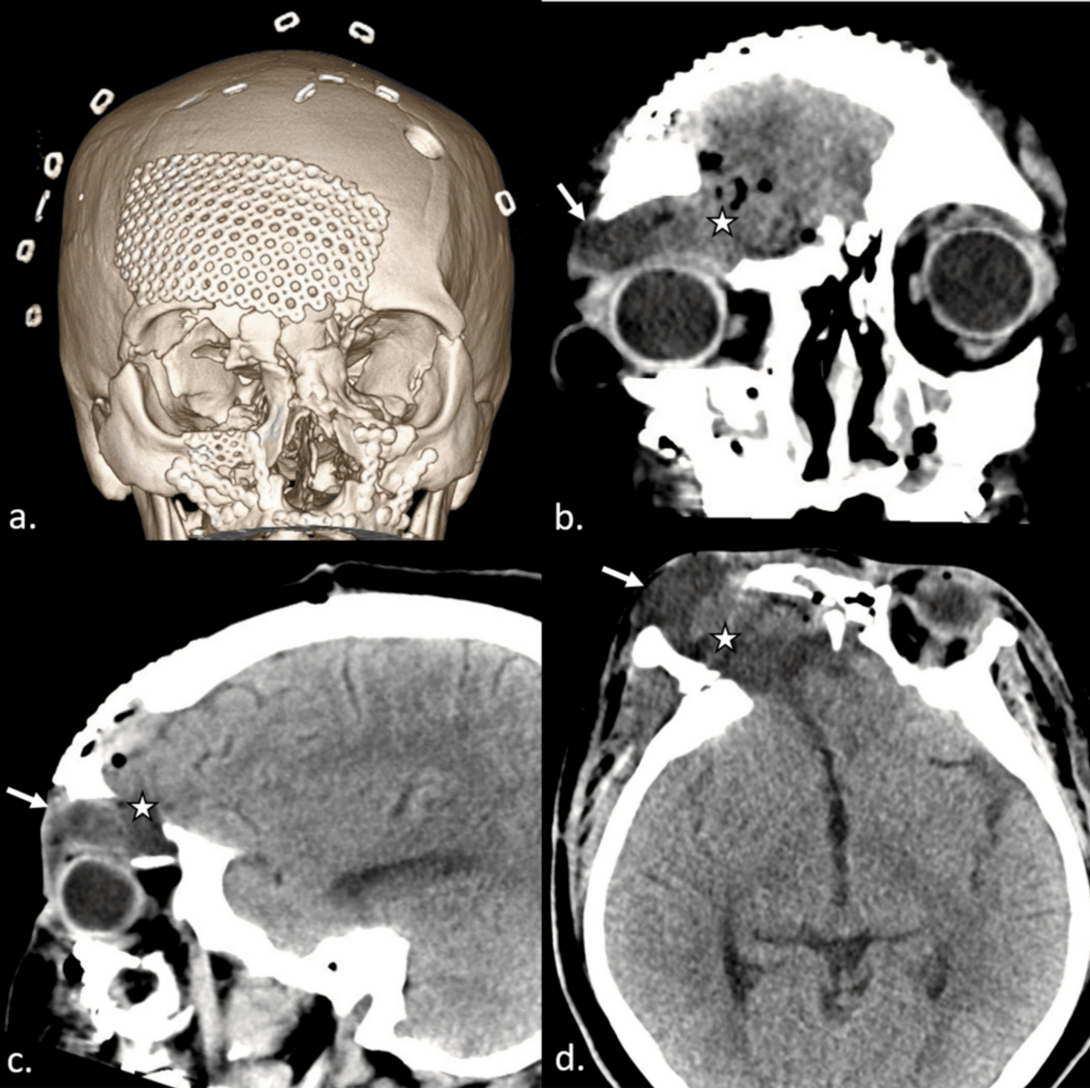
Further follow-up CT The 3D volume-rendered image (a) shows postsurgical changes after the revision of frontal cranioplasty with mesh placement to address the upper forehead aspect of the pseudomeningocele with a resolution of the pseudomeningocele along the forehead. Coronal, sagittal, and axial plane images (b, c, and d) demonstrate persistence, but the decreased size of the pseudomeningocele extending into the right superior orbit and periorbital space (white arrows) through a persistent defect in the right orbital roof (white stars). CT: computed tomography, 3D: three dimensional

Mesh cranioplasty and duraplasty addressed the anterior frontal skull base and frontal calvarial defects. CT head was repeated after the surgery, showing postoperative changes of anterior frontal mesh repair with a resolution of the fluid collection along the anterior frontal calvarial aspect but a persistent pseudomeningocele extending into the orbit and superior periorbital tissues through the defect in the orbital roof (Figure 3). The patient received instructions to follow up for a second-stage repair of the orbital roof.

## Discussion

An orbital pseudomeningocele is an extradural collection of CSF extending into the orbit and is a rare complication of an orbital roof fracture. In most reported cases, the pseudomeningocele is confined to the orbit [8-11]. However, we present a unique case where the pseudomeningocele extended superficially into the periorbital region, which has not been previously reported in the literature to our knowledge.

The clinical presentation and the onset of a traumatic orbital pseudomeningocele can vary. Patients may present with worsening pulsatile or non-pulsatile proptosis (with possible associated periorbital bulge as seen in our case), hypoglobus, diplopia, limited eye movement, and worsening visual acuity [6,8]. A thorough ophthalmological examination should be performed in such patients as part of a physical examination immediately following the trauma.

CT is the preferred modality for detecting orbital, skull base, and calvarial fractures and evaluating the orbital soft tissues in acute trauma [12]. It can also readily detect fluid collections, as in this case. In addition, the presence of pneumocephalus immediately adjacent to an orbital roof fracture can have high sensitivity for an associated dural laceration [13]. MRI has superior contrast resolution compared to CT. It can distinguish simple fluid collections like CSF from other types of fluid collections such as hemorrhage, and it may delineate herniated brain tissue if there is an encephalocele present [14]. Of note, there have been prior reports of a chronic pseudomeningocele becoming encysted within the orbit with the formation of a pseudo-membrane and the development of calcifications in its wall over time [10].

It is important to consider an orbital pseudomeningocele as a differential diagnosis in patients presenting with orbital roof fractures and periorbital swelling, as in our case. The extension of a pseudomeningocele into the periorbital region may be confused with other conditions, such as cellulitis or periorbital contusion, on physical examination [15]. Clinicians should maintain a high index of suspicion for pseudomeningoceles when evaluating these patients.

Managing a traumatic orbital pseudomeningocele requires a multidisciplinary approach involving neurosurgery, ophthalmology, and otolaryngology. Nonsurgical treatment for symptomatic patients might include head elevation and avoidance of activities increasing intracranial pressure, medication to reduce CSF pressure, or transcutaneous aspiration [6,8]. A lumbar drain may be placed for CSF diversion as a temporary treatment for a CSF leak to allow healing [16]. However, surgical treatment is required if nonsurgical measures fail or there is concern for significant complications such as vision loss; surgical management might include orbital decompression and repair of the skull base and dural defect [6,8,11].

A limitation of our study is the sparsity of research available regarding orbital pseudomeningoceles and their clinical effect. Ultimately, the prognosis depends on the severity of the trauma, prompt diagnosis, and the adequacy of therapy. Accurate imaging interpretation and characterization of the orbital trauma and complications play an essential role in timely diagnosis and directing appropriate management. Delayed and inadequate treatment can lead to persistent pseudomeningoceles from persistent CSF leaks and potentially permanent vision damage.

## Conclusions

A traumatic orbital pseudomeningocele is a rare complication of orbital roof fracture. Imaging with CT is key to evaluating for fractures and skull base defects and the presence and extent of a pseudomeningocele. Imaging assessment can also facilitate comprehensive preoperative planning. Our case contributes to the evolving understanding of traumatic orbital pseudomeningoceles, emphasizing the significance of diagnostic imaging and collaborative care for optimal patient outcomes.
